# The effects of Suramin on Ca^2+^ activated force and sarcoplasmic reticulum Ca^2+^ release in skinned fast‐twitch skeletal muscle fibers of the rat

**DOI:** 10.14814/phy2.13333

**Published:** 2017-07-25

**Authors:** Dane W. Williams, Dimitrie George Stephenson, Giuseppe S. Posterino

**Affiliations:** ^1^ Department of Physiology, Anatomy and Microbiology La Trobe University Melbourne Victoria Australia

**Keywords:** EC‐coupling, mechanically skinned, skeletal muscle, suramin

## Abstract

Suramin has long been used in the treatment of various human diseases. Intravenous infusions of Suramin are commonly administered to patients over extended periods of time but there are a number of significant contraindications with peripheral muscle weakness being one of the most frequently reported. Previous work has shown that even after a single infusion (300 mg kg^−1^) Suramin remains in skeletal muscle in effective concentrations (11.6 *μ*g mL ^−1^; 84 days) for prolonged periods. These observations provide a strong rationale for investigation of the specific effects of Suramin on skeletal muscle function. Single mechanically skinned fibers were directly exposed to Suramin (10, 100 or 500 *μ*mol L^−1^) for defined durations (2–10 min) in controlled physiological solutions that mimic the intracellular ionic environment of a fiber. Suramin treatment (10–500 *μ*mol L^−1^) directly affected the contractile apparatus in a dose‐dependent manner causing a decrease in Ca^2+^‐sensitivity (pCa50 = −log (Ca^2+^) concentration, where 50% of maximum Ca^2+^‐ activated force is produced) by 0.14 to 0.42 pCa units and reduction in maximum Ca^2+^‐activated force by 14 to 62%. Suramin treatment (100 *μ*mol L^−1^ for 10 min and 500 *μ*mol L^−1^ for 2 min) also caused development of a Ca^2+^‐independent force corresponding to 2.89 ± 4.33 and 16.77 ± 7.50% of pretreatment maximum Ca^2+^‐activated force, respectively. Suramin treatment (100 *μ*mol L^−1^, 2 min) also increased the rate of sarcoplasmic reticulum (SR) Ca^2+^ release without significant changes in SR Ca^2+^ uptake. We report new functional effects for Suramin related to alterations in both the contractile apparatus and SR Ca^2+^‐handling of skeletal muscle that may contribute to the peripheral muscle weakness noted in human pharmacological treatments.

## Introduction

Suramin is a broad acting polyanionic compound that has been used over many decades, initially for the treatment of African sleeping sickness (African trypanosomiasis) (Reincke et al. 1994; Joshi et al. 2005) and subsequently for river blindness due to onchocerciasis (Schulz‐Key et al. 1985). More recently it has also become a treatment modality for adults with recurrent high‐grade gliomas (Takano et al. 1994; Grossman et al. 2001). Suramin is a polysulfonated naphthyl urea that is typically administered i.v. (reported dosages vary), and less frequently by i.m. injection, on a weekly basis over a prolonged period of time, often several months. Investigations that focused on optimal in vivo concentrations for anticancer treatments found that 10% solution Suramin, with a *t*_1/2_ of 45–55 days, did not exceed 150 *μ*g mL^−1^ (104 *μ*mol L^−1^) on a weekly administration program (Van Rijswijk et al. 1992). This study reported an optimal serum concentration in the anticancer treatment of 319 *μ*g mL^−1^ (223 *μ*mol L^−1^). Due to global administration protocols and the broad acting nature of Suramin, a number of contraindications have been reported. Among the most significant side effects are severe fatigue, malaise and lethargy, which all may be manifest as a broad spectrum neuromuscular peripheral weakness of the hands, arms, legs and feet of patients (Eisenberger et al. 1995; Grossman et al. 2001). The skeletal muscle itself represents a large volume target for Suramin action, with the relative skeletal muscle mass of the body making up 30.6% ±5.5 of women and 38.4% ±5.1 in men (Janssen et al. 2000). Following a single dose of Suramin, persistently high intracellular concentrations have been observed in various tissues, notably the cortex of the kidney, spleen red pulp, bone marrow, and basement membranes surrounding bone, muscle groups and other organs (McNally et al. 2000). McNally and colleagues reported that after a single 1 h i.v. treatment of 300 mg kg^−1^ Suramin, muscle contained 94.9 *μ*g mL^−1^ of Suramin, a concentration that fell to 11.6 *μ*g mL^−1^ 84 days after treatment.

Suramin has been shown to act as an ATP antagonist for P2 purinoceptors (Nakazawa et al. 1990; Inoue et al. 1991; El‐Ajouz et al. 2012) and also as a strong activator of the Ryanodine Receptor (RyR), although at a site that is different from that for adenine nucleotide binding (Hohenegger et al. 1996; Sitsapesan and Williams 1996) possibly through an action on the Calmodulin (CaM) binding site of the RYR (Sitsapesan and Williams 1996; Klinger et al. 1999; Papineni et al. 2002; Hill et al. 2004). Therefore, the reduced muscular contractile ability or weakness described in patients treated with Suramin may be a consequence of Suramin directly affecting the Ca^2+^‐handling properties of the sarcoplasmic reticulum (SR). Suramin has been shown to also affect SERCA (Emmick et al. 1994) in addition to RyR. In addition, there is a distinct possibility that Suramin also affects the ability of the contractile apparatus to produce force, which has not previously been investigated.

In this study, we use the mechanically‐skinned skeletal muscle fiber preparation (Lamb and Stephenson 1990b) that retains a structurally intact SR and contractile apparatus to probe the effect of Suramin on the contractile apparatus and Ca^2+^‐release and uptake by SR over a wide, clinically relevant, concentration range (10–500 *μ*mol L^−1^). We show that Suramin treatment greatly affects both the Ca^2+^‐sensitivity and maximum Ca^2+^‐activated force and SR Ca^2+^ release through the RyR's. We hypothesize that as a result of Suramin treatment there will be changes in Ca^2+^‐sensitivity and maximum Ca^2+^‐activated force associated with direct effects on the contractile apparatus, as well as effects on the SR Ca^2+^ release through the RyR's. The combination of these actions helps to explain the Suramin‐induced peripheral muscle weakness noted in human pharmacological treatments.

## Methods

### Ethical statement

All experiments and procedures were approved by the La Trobe University Animal Ethics Committee (AEC) in accordance with the National Health and Medical Research Council (NHMRC) of Australia and ARRIVE guidelines.

### Animals and fiber preparation

A total of 24 male Long‐Evans hooded rats (*Rattus norvegicus*), obtained from Monash University and housed at La Trobe University ranging in age from 215 to 324 days were used in these experiments. Animals were kept at the La Trobe University Animal House, housed 3 to a standard cage in a pathogen‐free environment, kept at a constant temperature (23°C) with 12 h day‐night cycles and provided with water and standard pellet rat chow ad libitum.

#### Dissection

Rats were killed by an overdose of isoflurane (2% v v^−1^). The extensor digitorum longus (EDL) muscle was dissected out immediately postmortem, blotted on filter paper and pinned under paraffin oil (Ajax Chemicals, Sydney, Australia) at resting length on Sylgard 184 (Dow Chemicals, Midland, MI).

#### Fiber skinning and mounting

With the aid of a dissecting microscope (SMZ1000 or SMZ800, Nikon, Japan) fibers were isolated and mechanically skinned using a pair of fine forceps (Inox. No. 5, Dumont, Switzerland). As described by Fink and Stephenson (1987), skinned fibers were then tied at one end using fine silk suture and mounted to a force transducer (AME875 SensoNor, Horten, Norway) with the other end fixed using a mounted pair of forceps. This force transducer converted mechanical responses of fibers to electrical responses via a Wheatstone bridge circuit. Electrical responses were amplified and recorded in parallel on a chart recorder (Linear, Milpitas, CA) and Powerlab PC‐based data acquisition system (ADInstruments, Sydney, NSW, Australia) LabChart v7.0.3.

The resting length and diameter of the fiber (~40 *μ*m for all fibers) were then measured prior to application of a 20% stretch as previously described (Lamb and Stephenson 1990a). This length change adjusted the average sarcomere length of fibers, previously shown to result in a length of approximately 2.8 *μ*m (Stephenson and Williams 1982).

#### Randomization and blinding

After mounting, fibers were randomly selected to undergo a treatment or control protocol in all procedures. Single blinding protocols were implemented during the data acquisition phases with unlabeled fiber and solution data, ensuring values were obtained without the knowledge of what experimental protocol the fibers had been undertaken.

### Experimental procedures

All experiments were performed at room temperature (23 ± 2°C). All protocols utilized the force response of the skinned fiber segment as the primary measure of SR Ca^2+^‐ release and Ca^2+^‐sensitivity of the contractile apparatus.

#### Measurement of Ca^2+^‐sensitivity and maximum Ca^2+^‐activated force

Skinned fibers were immersed in a heavily Ca^2+^‐buffered K‐EGTA solution (relaxing solution) which contained (mmol L^−1^): EGTA, 50 mmol L^−1^; HEPES, 90 mmol L^−1^; Mg^2+^_total_, 10.3 mmol L^−1^ (free Mg^2+^, 1 mmol L^−1^); ATPtotal, 8 mmol L^−1^; creatine phosphate (PCr)total, 10 mmol L^−1^; K^+^, 125 mmol L^−1^; Na^+^, 36 mmol L^−1^; pH, 7.10. Fibers were then exposed to a series of equivalent EGTA buffered solutions with progressively higher concentrations of free calcium (expressed in pCa units (−log[Ca^2+^]) from >9 to 4.5) by mixing different ratios of the relaxing solution above with a Ca^2+^‐EGTA solution (pCa 4.5) (maximum‐activating solution) that was identical in composition to the relaxing solution with the exception that it contained (mmol L^−1^): Mg^2+^total, 8.12 mmol L^−1^ (free Mg^2+^, 1 mmol L^−1^); Ca^2+^_total_, 49.7 mmol L^−1^. This resulted in the production of increasing force responses that plateaued once maximum Ca^2+^‐activated force was achieved at saturating free [Ca^2+^] resulting in the identification of the force‐Ca^2+^ relationship for the fiber (force‐pCa; where pCa is the −ve log_10_ of the free [Ca^2+^]). Once the maximum Ca^2+^‐activated force of fibers was confirmed, fibers were relaxed by re‐immersion in the relaxing solution. This activation sequence was repeated three times and fibers were only used if the maximum Ca^2+^‐activated force response in a sequence did not decrease by more than 5% between repetitions. Fibers were then incubated in a relaxing solution containing Suramin (100 *μ*mol L^−1^ for 2 or 10 min or 500 *μ*mol L^−1^ for 2 min). Fibers were then washed in fresh relaxing solution for 10 min to remove residual Suramin and the activation protocol was repeated three more times. The average of the force responses from the three activations was then used to construct a composite force‐pCa curve for each experimental group. For each of the three repetitions (both before and after Suramin treatment), the steady‐state submaximal force responses were normalized to its own maximum Ca^2+^‐activated force (considered 100%) and then the average of these three sets of values were used to construct a single composite force‐pCa curve which was represented graphically using GraphPad Prism v5.03. The pCa50 values (derived using equation (1)) and the maximum Ca^2+^‐activated force was compared to the control (before treatment) force‐pCa relationship.

(1) Y=Bottom+(Top-Bottom)/(1+10^((LogEC50-X)∗Hillslope))

where: *Y* = *Y*‐axis; Bottom = Bottom Plateau; Top = Top Plateau; EC_50_ = *X* value corresponding to half‐maximal (*Y*) response; *X* = *X*‐Axis; HillSlope = Gradient at *P*_50._

#### Measurement of caffeine‐induced Ca^2+^ release from the SR

A qualitative assessment of the amount of releasable Ca^2+^ and properties of SR Ca^2+^ release from the SR of skinned fibers was determined by examining the caffeine‐induced force response. The area under a caffeine‐induced force transient has been previously shown to be proportional to the SR Ca^2+^ content over a range of SR [Ca^2+^] (Endo and Iino 1980; Fink and Stephenson 1987; Launikonis and Stephenson 1997). Initially, freshly skinned fibers were separated into two experimental groups, control and Suramin treatments. In the control group, fibers were first equilibrated in a K‐HDTA solution mimicking the normal ionic intracellular milieu. While it is likely that weakly bound and free CaM to have diffused out of the fibers, no exogenous CaM was added to the solutions. The solutions contained (mmol L^−1^): HEPES, 90; hexamethylenediamine‐tetraacetate (HDTA) 50; EGTA, 0.05; Mg^2+^_total_, 8.5 (free Mg^2+^, 1); ATP_total_, 8; PCr_total_, 10; K^+^_total_, 125; Na^+^_total_, 36; pH 7.10) and fibers were bathed for 2 min and then briefly equilibrated in an identical solution (the wash solution; which contained 1 mmol L^−1^ total EGTA (pCa > 9), for 6 sec) before the SR was emptied of all releasable Ca^2+^ by exposure to a 30 mmol L^−1^ caffeine solution (30 mmol L^−1^ caffeine dissolved in a low (0.13 mmol L^−1^) [Mg^2+^_free_] equivalent of the K‐HDTA solution. This caffeine solution rapidly empties the SR of all stored Ca^2+^ (Fryer and Stephenson 1996). Fibers were then washed for 1 min in the wash solution to ensure the SR could not be refilled with Ca^2+^ during this time and then fibers were equilibrated in an equivalent solution (pCa 6.7; 1 mmol L^−1^ total EGTA) for defined time periods (10, 20, 30, 60, and 120 sec) to precisely refill the SR with varying amounts of Ca^2+^. In between consecutive load periods, fibers were briefly washed (10 sec) in the wash solution, a process that stops further SR Ca^2+^ loading. Fibers were then transferred back to the 30 mmol L^−1^ caffeine solution triggering complete Ca^2+^ emptying of the SR. The ensuing area of the caffeine‐induced force transient was subsequently recorded. After the last loading period in the sequence described above (120 sec load), fibers were then washed for 12 min in the wash solution to account for the Suramin treatment time (see below) and then the SR was depleted again with 30 mmol L^−1^ caffeine solution to empty the SR of all Ca^2+^ before the load protocol (as above) was repeated for each loading period (10–120 sec) once more. This was done to test for reproducibility of the SR Ca^2+^‐ loading protocol over time. In separate fibers, this same procedure was repeated with the exception that after the initial control load curve was established, fibers were treated with 100 *μ*mol L^−1^ Suramin (added to the wash solution) for 2 min followed by a further 10 min wash in a different wash solution to remove Suramin. A second load protocol was then repeated.

#### Conversion of force transients to free calcium estimates

**Figure 1:**
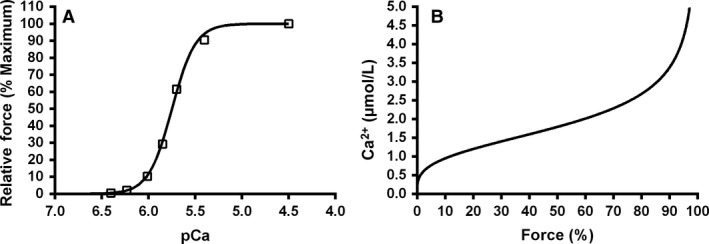
(A) Representative relationship of relative force (percentage of maximal activated force) as a function of calcium concentration (pCa). (B) Mathematical derivation from force‐pCa curve of the Ca^2+^‐force relationship.

To distinguish between the potential direct effects of Suramin on the pharmacological release of SR Ca^2+^ by caffeine and any concomitant Suramin‐induced alterations in Ca^2+^ sensitivity and/or maximum Ca^2+^‐activated force (see below), it was necessary to estimate the profile of the Ca^2+^‐ change underlying the caffeine‐induced force response. The graphical relationship between force and the activating Ca^2+^ concentration represents a force‐pCa relationship as shown in Figure 1A. This relationship can be used to mathematically derive a relationship (Ca^2+^‐force graph; Fig. 1B) that allows for determination of the Ca^2+^ concentration, this will account for the possibility that Suramin will affect the calcium sensitivity of each fiber and is required for each of the force transients generated by the pharmacological release (e.g., caffeine) of SR Ca^2+^ of the same fiber using the following equation

(2) $$\left[{\text{Ca}}^{2+}\right]=n\sqrt{\left(P/P_{\max}\right)\left(1-P/P_{\max}\right)}\times 10^{-{\text{pCa}}_{50}}$$

This equation is a derivative of that previously devised by Rees and Stephenson (1987)

(3) $$P/P_{\max}={\left[{\text{Ca}}^{2+}\right]}^{n}/\left({\text{pCa}}_{50}^{n}+{\left[{\text{Ca}}^{2+}\right]}^{n}\right)$$

where: *n *= average Hill coefficient (Hill slope) derived from corresponding force‐pCa curve; *P/P*_max_ = Force (*t*)/Maximum Force (*t*) at each time point (*t*) of the force transient; pCa50 = Log of Calcium concentration at 50% of maximum force derived from the corresponding force‐pCa curve.

All force transients were then converted into estimated free Ca^2+^ traces by reference to the Ca^2+^‐force curve (Fig. 1B). From these free Ca^2+^ estimates the following parameters were quantified: (1) peak free calcium concentration (*μ*mol L^−1^) during caffeine‐induced Ca^2+^‐release from the SR, (2) maximum rate of Ca^2+^ rise (nmol ms^−1^) calculated as the rate of Ca^2+^ rise from the slope 20 to 80% of the peak Ca^2+^ transient, as an indicator of the rate of Ca^2+^‐release from the SR (the rates for different treatments were then expressed as a percentage of the 120 sec control) and (3) the Area Under the Curve of the resultant Ca^2+^ estimates associated with the caffeine‐induced Ca^2+^‐release as an indicator of total amount of SR Ca^2+^ content (Endo and Iino 1980; Fink and Stephenson 1987; Launikonis and Stephenson 1997.

#### Exclusion criteria

Throughout testing and analysis a number of criteria were needed to be met to ensure continued usage of the sample and/or data these are listed for each procedure below:

1. Damage to the muscle fiber before or during experimental preparation and procedures.
2. Fibers during initial baseline measurements recorded an equal or >5% decrease in total force production.

### Normalization and statistical comparison

Data were normalized to account for any unwanted variation between samples. Curve Fitting parameters included ensuring *Y*_max_ was 100, which lead to a Standard Deviation of zero. This was done to ensure accurate comparisons between the other variables.

Data are presented as means ± SD, and/or actual values where appropriate. *n* denotes the number of fibers used. GraphPad Software (Prism, San Diego, CA) was used for statistical analysis and curve fitting. Student *t*‐tests (paired, two tailed) were used to compare the means of the control and test groups. Lines of best fit to Ca^2+^ loading time‐dependent parameters were plotted using the following equation:

$Y=Y_{\max}\left(1-A^{\left(-K\times X\right)}\right)$. Significance was indicated when *P* < 0.05 and denoted with an asterisk (*) for all statistical analysis where multiple analyses are carried out additional significance denoted with a hash (#).

## Results

### Effects of Suramin on the contractile apparatus of fast‐twitch skeletal muscle fibers

**Figure 2:**
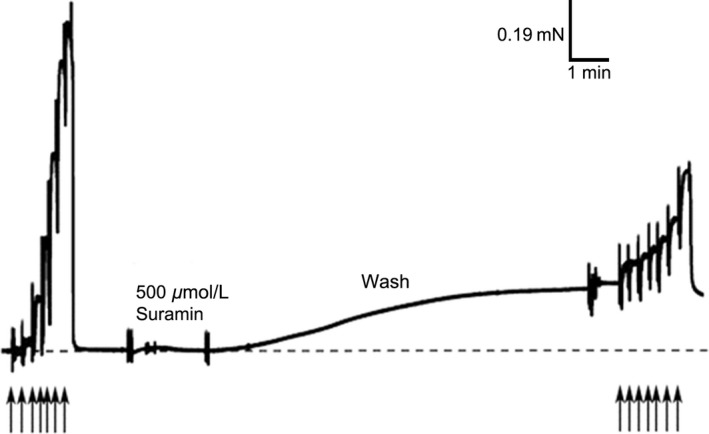
Representative force recording of the change in force level in an EDL mechanically skinned muscle fiber following activation in solutions of different pCa before and after a 2 min exposure to 500 *μ*mol L^−1^ Suramin. Each consecutive arrow indicates a change to a solution with a higher free [Ca^2+^] (lower pCa) (pCa values from left to right 8.40, 6.40, 6.22, 6.02, 5.88, 5.75, 5.48, and 4.50). [Ca^2+^] was increased until a stable maximum force level was achieved. Note the depression of Ca^2+^‐activated force levels following Suramin treatment. The significant elevation in resting force (during the wash period) and the depression of Ca^2+^‐activated force levels following Suramin treatment were comparable to longer treatment at 100 *μ*mol L^−1^ Suramin.

Figure 2 shows the force‐pCa relationship for a single mechanically skinned muscle fiber before and after exposure to 500 *μ*mol L^−1^ Suramin for 2 min. Following a 2 min exposure to Suramin (500 *μ*mol L^−1^) in a relaxing solution, and during the 10 min wash period in the “wash solution” (relaxing solution pCa >9) a pronounced Ca^2+^‐independent force developed. Subsequent determination of the force‐pCa relationship also revealed a marked decrease in the Ca^2+^‐activated force across the entire pCa range.

**Figure 3:**
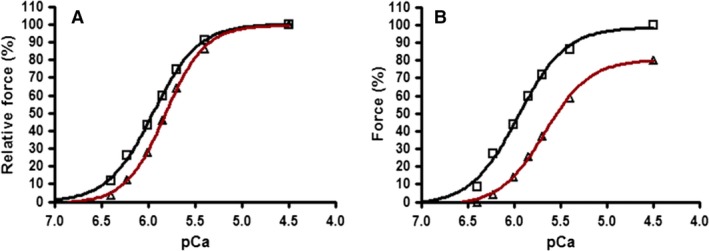
Force‐pCa relationships for individual extensor digitorum longus mechanically skinned muscle fibers exposed to 10 *μ*mol L^−1^ Suramin (2 min). (A) Changes in fiber sensitivity to Ca^2+^ occurring from Suramin exposure. All force values are represented as a percentage of force produced at pCa 4.5 in at either the control or Suramin protocol. (B) Change is absolute force following Suramin exposure. All values are represented as the percentage relative to pCa 4.5 pre‐treatment (Control). Control values denoted as □; Suramin treatment denoted as ▵.

The effect of Suramin on the graphical relationship between force and the activating Ca^2+^ concentration in a single fiber treated with 10 *μ*mol L^−1^ for 2 min is presented in Figure 3. Table 1 compares the measured parameters of the fitted force‐pCa curves (pCa50, Hill slope, Maximum Ca^2+^‐Active Force and “Resting” Force [force in relaxing solution of pCa > 9]) for the four variations of Suramin treatment shown. All four treatment groups showed a statistically significant decrease in Ca^2+^‐sensitivity (which is an increase in the antilog of the free [Ca^2+^] indicated in the table by −pCa50 values). Fibers treated with 10 *μ*mol L^−1^ Suramin showed a significant change in pCa50 with an average decrease in 0.14 ± 0.11 (*n* = 5). The group treated with 100 *μ*mol L^−1^ Suramin for 2 min also had a significant change in the pCa50 value with an average decrease in 0.18 ± 0.09 (*n* = 9). Fibers treated with 100 *μ*mol L^−1^ Suramin for 10 min in addition to an average decrease in the pCa50 of 0.36 ± 0.19 also showed a decrease in the Hill slope co‐efficient of 2.67 ± 0.76 (*n* = 10) and the presence of Ca^2+^‐independent force (~3% of the maximum Ca^2+^‐activated force). Fibers treated with 500 *μ*mol L^−1^ Suramin (2 min) showed further significant decreases in both pCa50 and the Hill slope co‐efficient of 0.42 ± 0.13 and 1.11 ± 0.83 (*n* = 11), respectively, and the presence of considerable Ca^2+^‐independent force of ~16% of the maximum Ca^2+^‐activated force.

**Table 1:** The effect of Suramin on Ca^2+^‐activated force on mechanically skinned fast‐twitch skeletal muscle fibers

	δpCa50	δ*n*(Hill slope)	δMaximum Ca^2+^‐activated force (%)	Resting force (%)	Sample size (*n*)
10 *μ*mol L^−1^ Suramin (2 min)	−0.14 ± 0.11^1^	−0.11 ± 0.34	−14.00 ± 7.16^a^	0.00 ± 0.00	5
100 *μ*mol L^−1^ Suramin (2 min)	−0.18 ± 0.09^a^	+0.13 ± 0.51	−18.88 ± 15.96^a^	0.00 ± 0.00	9
100 *μ*mol L^−1^ Suramin (10 min)	−0.36 ± 0.19^a^	−2.67 ± 0.76^a^	−37.4 ± 38.93^a^	+2.89 ± 4.33^a^	10
500 *μ*mol L^−1^ Suramin (2 min)	−0.42 ± 0.13^a^	−1.11 ± 0.83^a^	−61.81 ± 13.83^a^	+16.05 ± 7.43^a^	11

Comparative Control Mean Values; Sample Size = 35, pCa50 = 5.82 ± 0.12, *n* = −3.13 ± 0.24, Max = 0.42 mN ± 0.18.

a Indicates significance versus control all data are presented relative to control values in the same fibers. For example a negative *n*(Hill slope) means that the respective curve is steeper than for controls.

The maximum Ca^2+^‐activated force was also significantly affected by Suramin treatment (Table 1). Fibers treated with 10 *μ*mol L^−1^ Suramin (2 min) showed a decrease in the maximum force by 14 ± 7.16% compared to the control. This decreased further by 18.9 ± 15.96% in fibers treated with 100 *μ*mol L^−1^ Suramin (2 min) and by 37.4 ± 38.93% after a 10‐min treatment. Muscle fibers treated with 500 *μ*mol L^−1^ Suramin for 2 min exhibited the greatest reduction in maximum active force with an average reduction in 61.8 ± 13.83%. These data reveal that Suramin has a significant effect on both the Ca^2+^‐activated force and Ca^2+^‐independent (resting) force exerted through a direct effect on the contractile apparatus. The significant reduction in both maximum Ca^2+^‐activated force and the development of Ca^2+^‐independent force after Suramin treatment, suggests that Suramin potentially removes key regulatory proteins of the contractile apparatus – the troponin complex.

### Addition of exogenous Troponins to Suramin‐treated fibers

To test whether the changes in both Ca^2+^‐dependent and independent force observed after Suramin treatment are due to removal of the Troponin complex, a subset of Suramin treated fibers were immersed in a relaxing solution that contained equimolar concentrations (5 mmol L^−1^) of Troponin I, Troponin T and Troponin C rabbit proteins after we first checked on three control fibers that such troponin treatment did not affect the force responses in these fibers.

Table 2 compares the relative force‐pCa relationships of mechanically skinned fibers where the Troponin mixtures were reintroduced following the Suramin treatment (100 *μ*mol L^−1^, 10 min treatment, 10 min wash and 500 *μ*mol L^−1^ Suramin, 2 min treatment and 10 min wash). Troponin re‐addition did not reverse the changes in Ca^2+^ sensitivity caused by Suramin (100 or 500 *μ*mol L^−1^), but was able to reverse a significant component of the reduction in the Hill slope of the force‐pCa curve caused by 100 *μ*mol L^−1^ Suramin application. Similarly, troponin re‐addition partially restored the diminished capacity of fibers to generate Ca^2+^ activated force following 100 *μ*mol L^−1^ Suramin treatment and significantly reduced the Ca^2+^‐independent force generated by both Suramin treatments by ~50%.

**Table 2:** Collated data for the effects on Ca^2+^‐activated force of Troponin re‐addition after Suramin treatment of mechanically skinned fast‐twitch skeletal muscle fibers

	δpCa50	δ*n*(Hill slope)	δMaximum Ca^2+^‐activated force (%)	Resting force (%)	Sample size (*n*)
100 *μ*mol L^−1^ Suramin (10 min)	−0.33 ± 0.10^a^	−1.31 ± 0.39^a^	−52.47 ± 20.67^a^	4.81 ± 4.78^a^	6
Troponin added	−0.27 ± 0.07^a^	−0.81 ± 0.39^a^^,^ b	−32.39 ± 15.75^a^^,^ b	2.32 ± 2.52^a^^,^ b	
500 *μ*mol L^−1^ Suramin (2 min)	−0.40 ± 0.11^1^	−1.24 ± 0.85^a^	−64.25 ± 10.92^a^	16.77 ± 7.50^a^	8
Troponin added	−0.37 ± 0.14^a^	−1.15 ± 0.91	−51.34 ± 16.38^a^	7.09 ± 12.56^b^	

a Indicates significance versus control.

b Indicates significance versus Suramin treatment. All data are presented relative to control values in the same fibers. For example a negative *n*(Hill slope) means that the respective curve is steeper than for controls.

### The effects of Suramin on the calculated calcium movements from the SR of mechanically skinned single EDL muscle fibers

**Figure 4:**
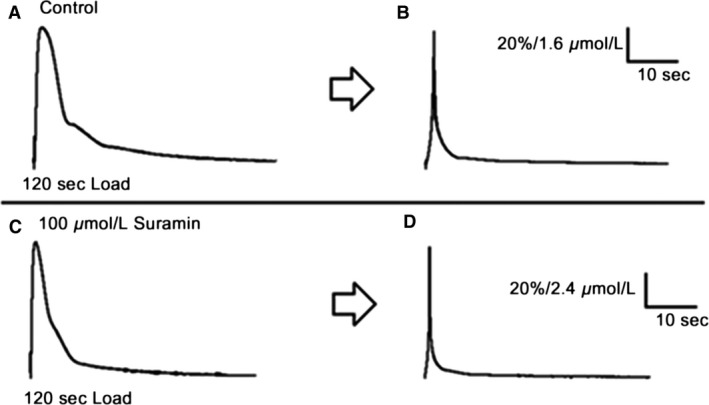
The conversion of representative force transients (% of maximum) into free Ca^2+^ transients for a single mechanically skinned extensor digitorum longus muscle fiber after 120 sec Ca^2+^ loading of the SR. Representative force transients for (A) control and (C) post–Suramin treatment (100 *μ*mol L^−1^, 2 min) conditions. The calculated free calcium transients (*μ*mol L^−1^) for (B) control and (D) force transients, using the conversion equation (1).

**Figure 5:**
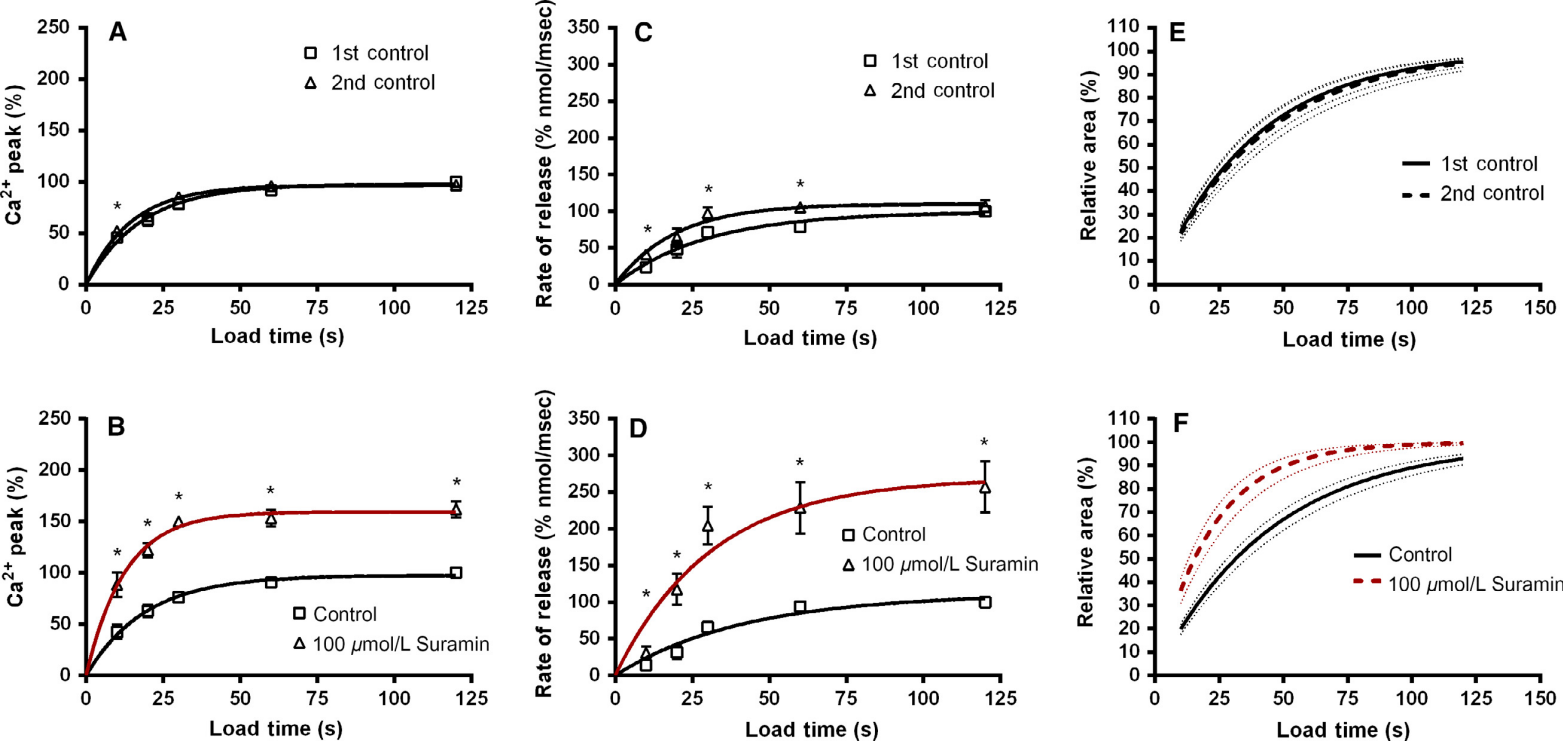
Suramin effects on key characteristics of Ca^2+^‐transients derived from caffeine‐induced force transients in single mechanically skinned extensor digitorum longus muscle fibers. (A and B) Peak concentration of Ca^2+^ transients (% of 120 sec Control); (C and D) Rate of change in Ca^2+^ (measured 20–80% of peak level) (% of 120 sec Control, measured as mol s^−1^); and (E and F) The relative area under free calcium transients. (A, C and E) Control (no Suramin); (B, D and F) 100 *μ*mol L^−1^ Suramin (2 min). Results were obtained from six rats.

Figure 4 shows the conversion of recorded force responses into Ca^2+^ transients as indicated in the Methods (Equation (1) and Fig. 1B). Panels A and C show force transients for caffeine‐induced Ca^2+^ release after 120 sec of Ca^2+^ loading before and after exposure to 100 *μ*mol L^−1^ Suramin for 2 min, respectively and Panels B and D illustrate the free Ca^2+^ estimates. Figure 5 compares collated data for caffeine‐induced Ca^2+^‐release force responses converted into free Ca^2+^ transients. Panels A & B show the peak (% of 120 sec control) of the free Ca^2+^ transients for control and Suramin treated fibers, respectively, C & D show the rate of Ca^2+^ rise (% nmol ms^−1^) called “rate of release” measured between the period 20 through to 80% of peak, and E & F show the area under the free Ca^2+^ transient (a reflection of the total amount of Ca^2+^ released from the SR (Endo and Iino 1980; Fink and Stephenson 1987; Launikonis and Stephenson 1997).

#### Peak free calcium concentration during release from the SR

Figure 5A shows the effect of a 12 min wash period between two sets of load‐deplete protocols in control fibers. It can be seen that the peak calculated Ca^2+^‐transient remains unchanged by the wash period. Figure 5B shows the effects of a Suramin treatment (100 mol L^−1^, 2 min, followed by 10 min wash) produced a significantly higher peak Ca^2+^‐transient at all loading times compared with the preceding control. Suramin caused an increase in the peak Ca^2+^‐transient by approximately twofold across the entire load curve.

#### The rate of calcium release from the SR

The rate of Ca^2+^ release from the SR under control conditions significantly increased by a small amount after the 12 min control wash period (Fig. 5C). However, in fibers treated with Suramin (100 *μ*mol L^−1^, 2 min; Fig. 5D) the rate of Ca^2+^ release significantly increased across all loading times by a much greater extent (>2 fold) compared to the control.

#### The relative area of free calcium estimates

Unlike the peak and the rate of rise of the Ca^2+^‐transient described above, which are affected directly by any change in RyR channel activity, the relative area under the caffeine‐induced Ca^2+^‐transient provides (see Methods) information about both the amount of Ca^2+^ loaded into the SR and thus an indication of the activity of the SR Ca^2+^‐ATPase (SERCA) and the total amount of Ca^2+^ subsequently released from the SR. Using the equation $Y=Y_{\max}\left(1-A^{\left(-K\times X\right)}\right)$ to plot a line of best fit through all loading times, we could assess the amount of Ca^2+^ loaded into the SR in control and Suramin treated fibers (Fig. 5E and F). It is important to note that loading in these experiments occurs after Suramin treatment and washout, which differs from the study by Emmick et al. (1994) in which Suramin was present. Thus we expect that little effect of Suramin on the SR Ca^2+^‐ATPase and thus the amount of Ca^2+^ loaded into the SR. Under both control conditions (Fig. 5E) and in fibers treated with 100 *μ*mol L^−1^ Suramin for 2 min (Fig. 5F) there was no significant difference in the curves showing the average line of best fit, indicating that Suramin did not alter the relative amount of Ca^2+^ in the SR. The mean data for these experiments is summarized in Table 3.

**Table 3:** Summary of curve‐fitting parameters in Figure 5E and F

$Y=Y_{\max}\left(1-A^{\left(-K\times X\right)}\right)$	Control	100 *μ*mol L^−1^ suramin	1st control	2nd control
*Y*_max_	100 ± 0.00	100 ± 0.00	100 ± 0.00	100 ± 0.00
*A*	1.23 ± 0.32	1.43 ± 1.05	1.00 ± 0.25	1.25 ± 0.51
*K*	0.03 ± 0.03	0.06 ± 0.05	0.03 ± 0.03	0.03 ± 0.03

## Discussion and Conclusions

### Effects of Suramin on myofibrillar components

A novel finding of this study was the marked effect of Suramin on the functions of the contractile apparatus and Ca^2+^‐regulatory system. Even at the lowest concentration used in this investigation (10 *μ*mol L^−1^ for 2 min) the maximum Ca^2+^‐activated force elicited at pCa 4.5 (see Table 1) was reduced by 14% and the sensitivity to Ca^2+^ decreased by 38% (δpCa50 = −0.14). Furthermore, at higher Suramin concentrations (100–500 *μ*mol L^−1^ Suramin), the maximum Ca^2+^‐activated force was decreased by 62%, the Ca^2+^‐sensitivity was decreased by a factor of 2.3–2.6 (δpCa50 = −0.36 to −0.42), and in addition, treated fibers developed a Ca^2+^‐independent force (termed “resting force” – see Table 1) that was 17% of the maximum Ca^2+^‐activated force. Subsequent treatment of fibers with a mixture of exogenous Troponins (Troponin C, I and T), partially reversed the effects of Suramin observed on resting force, maximum force and Hill slope. This suggested that the effects of Suramin on the contractile apparatus were partially mediated through the removal of Troponin C and Troponin I. Removal of Troponin C is consistent with the reduction in the Ca^2+^‐activated force. Given that Suramin has previously been reported to remove CaM from the RyRs (Klinger et al. 1999; Papineni et al. 2002), it is not surprising that it also removes Troponin C considering that Troponin C is a CaM‐like protein. Removal of Troponin I is consistent with an increase in the Ca^2+^‐independent force as occurs following treatment of muscle fibers with a highly negatively charged molecule, such as Orthovanadate (Allhouse et al. 1999).

### Effect of Suramin on SR Ca^2+^‐handling

The mechanically‐skinned fiber retains an intact and functional SR (Lamb and Stephenson 1990b). The properties of the SR Ca^2+^ uptake via the SR Ca^2+^‐ATPase and Ca^2+^ release from the SR via the RyRs were investigated using the contractile apparatus as an indicator of the cytoplasmic free Ca^2+^ movements. Treatment of skinned fibers with 100 *μ*mol L^−1^ Suramin for 10 min produced a marked increase in the rate of caffeine‐induced Ca^2+^ release at all levels of SR Ca^2+^ loading (see Fig. 5D) indicating the Suramin treatment clearly affected the RyRs in our preparation. It is worth noting that in this study Suramin was washed from the fibers following the 10 min treatment period and therefore, the observed effects of Suramin described in this study must result from some irreversible action of Suramin on the RyRs (in addition to the contractile myofibrillar components described earlier). Suramin is thought to compete with CaM on the RyR displacing CaM from this site (Klinger et al. 1999; Papineni et al. 2002; Sigalas et al. 2009). If this were the case, then our results are most likely explained by an increase in the open probability when activated following the removal of CaM from the RyRs as reported in other studies (Hill et al. 2004).

In our study calcium, loading by the SR was not affected after exposure and washout of Suramin suggesting that the SR Ca^2+^‐ATPase was not affected. A previous study by Emmick et al. (1994) reported the SR Ca^2+^‐ATPase was reduced in the presence of Suramin. Taken together, the results indicate that the Suramin effect on the SR Ca^2+^‐ATPase is fully reversible. Indeed, this was also observed by Sigalas et al. (2009) who showed that the SR Ca^2+^‐ATPase in permeablized cardiac cells was not affected by Suramin treatment for a similar time.

### Clinical relevance

This study demonstrates that Suramin does not only contribute to muscle weakness by affecting the properties of the RyRs but also has a strong lasting effect on skeletal muscle myofibrillar components which markedly reduces the ability of muscle to produce Ca^2+^‐activated force. Prolonged Suramin treatment also increased the level of Ca^2+^‐independent (resting) force which would contribute to an elevated resting cellular ATPase activity that would further lead to a generalized metabolic fatigue.

## Conflict of Interest

None declared.
